# Recognition of Ultrasound Artifact Mimicking Pulmonary Artery Dissection in Patients with Heart Disease

**DOI:** 10.1155/2019/4919416

**Published:** 2019-06-19

**Authors:** Weichun Wu, Na Zhang, David H. Hsi, Lili Niu, Yong Jiang, Yang Wang, Zhenhui Zhu, Hao Wang

**Affiliations:** ^1^Department of Echocardiography, Fuwai Hospital, National Center for Cardiovascular Diseases, China; ^2^Department of Function, The Second Central Hospital of Baoding City, Zhuozhou 72750, China; ^3^Department of Cardiology, Stamford Hospital, Stamford, Columbia University College of Physicians & Surgeons, CT, USA

## Abstract

**Purpose:**

Imaging artifacts are frequently encountered when performing clinical echocardiography. Based on our review of the literature, two-dimensional linear artifacts are mainly reported in the ascending aorta in patients with suspected aortic dissections. However, pulmonary artery artifacts that mimic pulmonary artery dissection have not been discussed. We herein report our experience with children and adults with preexisting heart conditions and pulmonary artery imaging artifacts.

**Methods:**

The study population comprised 10 patients with heart disease who were treated at our hospital from March 2015 to September 2017. Nine patients were children with congenital heart disease, mainly patent ductus arteriosus (n = 8), and one patient was an adult with pulmonary artery hypertension. Transthoracic echocardiography was performed in all patients.

**Results:**

We confirmed the diagnosis in six patients during a surgical operation for other indications and in four patients by computed tomographic pulmonary angiography. The most common pulmonary imaging artifact was observed from the left high parasternal view (9/10, 90%). Most of the artifacts were diagonally oriented (8/10, 80%), and a few were horizontally oriented. Half of the artifacts were located in the main pulmonary arteries with mild pulmonary artery dilatation. Pulmonary hypertension was seen only in the adult patient. The thymus gland was clearly seen in young patients.

**Conclusion:**

Pulmonary artery imaging artifacts in patients with preexisting heart disease during echocardiographic examination can mimic pulmonary artery dissection. Understanding the types and origins of these ultrasound artifacts is important to avoid a false-positive diagnosis.

## 1. Introduction

Echocardiography is the diagnostic method of choice to evaluate cardiac anatomy and physiology. However, imaging artifacts are frequently present in clinical practice [1–3]. Artifacts are images that are not real; are located in the wrong place; have an inappropriate brightness, shape, or size; or represent structures that are missing. Although modern ultrasound technology has reduced the presence of artifacts, we may still encounter some unusual cases that pose challenges to our clinical management of patients [4, 5]. Some reports have described artifacts of visceral and cystic structures [6, 7]. Imaging artifacts on echocardiography mainly occur in the ascending aorta in patients with suspected aortic dissections [8]. Other echocardiography artifacts have been misdiagnosed as left atrial appendage thrombi [9] or have presented as a mirror image [10] from a left atrial line mimicking a catheter in the left ventricle during transesophageal echocardiography. Transesophageal echocardiography and three-dimensional echocardiography can improve the image quality but may still produce artifacts [11]. We have encountered no previous reports of artifacts in the pulmonary artery by echocardiography in children and adults with heart disease.

## 2. Methods

From March 2015 to September 2017, we identified 10 patients with underlying heart disease in our hospital. The patients comprised nine females and one male aged 6 months to 37 years (mean, 5.98 ± 11.03 years). There were nine pediatric patients (age of 6 months to 6 years) with congenital heart disease and one female adult patient (age of 37 years) with systemic lupus erythematosus and pulmonary artery hypertension (PAH).

### 2.1. Echocardiography

Transthoracic echocardiography was performed in the supine and left lateral positions using a Philips IE33 system (Philips Healthcare, Amsterdam, The Netherlands) or GE Vivid 7 system (GE Healthcare, Chicago, IL, USA) with a 3.5- to 8.0-MHz phased-array transducer. All patients underwent standard two-dimensional and Doppler echocardiographic examinations, which included the suprasternal left heart long-axis view, aortic short-axis view, pulmonary artery long-axis view, and apical four-chamber view and the subxiphoid view of the pulmonary artery. In addition, we used special views to show the pulmonary artery, such as the high parasternal view, suprasternal aortic short-axis view, and pulmonary artery short-axis view. We analyzed artifacts including location, length, form, movement, degree of clarity, dilation of the pulmonary artery, and pulmonary pressure.

### 2.2. Surgical Procedures and Computed Tomography Scans

We gathered patient data including the operations and other diagnostic examinations performed in our hospital. We identified 18 patients with artifacts within the 2.5-year study period, but the artifacts in only 10 of these patients were confirmed by surgery (6 patients) and computed tomographic pulmonary angiography (CTPA) (4 patients).

To confirm artifacts or dissection, CTPA was performed using a Somatom Definition Open 64 CT scanner (Siemens, Munich, Germany). We acquired CTPA data during an intravenous injection of 50 ml of the iodinated contrast agent iomeprol (Iomeron 400; Bracco Imaging, Konstanz, Germany) at a rate of 5 ml/s and 20 ml of saline at a rate of 4 ml/s as a chasing bolus. The delayed scan time was set using the automatic trigger scanning mode; the threshold was 80 HU, and the region of interest was the pulmonary artery.

Six patients underwent patent ductus arteriosus (PDA) ligation or ventricular septal defect repair. The pulmonary arteries were inspected in the operating room if pulmonary artery artifacts were reported.

### 2.3. Statistical Analysis

Statistical analysis was performed with the SPSS 20.0 software package (IBM Corp., Armonk, NY, USA). Continuous variables are presented as mean ± standard deviation, and categorical variables are presented as number (percentage).

## 3. Results

The patients' demographic data are listed in Table 1. Notably, nine patients were female and nine were children. The patients comprised nine pediatric patients with congenital heart disease, mainly PDA (n = 8), and one female adult patient with systemic lupus erythematosus and PAH. Linear artifacts in the pulmonary artery imitating dissection were seen in these patients.

The most common pulmonary imaging artifact was observed in the left high parasternal view (9/10, 90%), and other artifacts were often seen in the aortic short-axis and pulmonary artery long-axis views (7/10, 70%). Seven artifacts were long (≥2 cm) and three artifacts were short (<2 cm). Most of the artifacts were diagonally oriented (8/10, 80%). All diagonal artifacts were long segments (100%). The linear artifacts almost always moved with the patient's respiration, most of them changed in different echocardiographic views, and some of them extended outside the pulmonary artery or heart. Half of the artifacts were located in the pulmonary arteries with mild pulmonary artery dilatation. Most of the patients had no PAH (9/10, 90%) according to the echocardiographic pressure measurement and clinical diagnosis. However, the one adult patient with PAH and a dilated pulmonary artery had imaging artifacts.

### 3.1. Echocardiography Case Presentation and Imaging Characteristics

**Figure 1:**
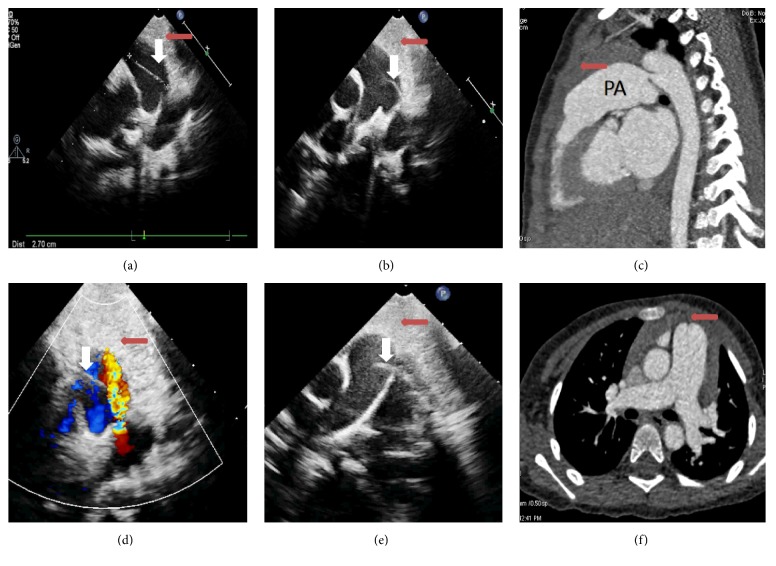
(a, b) Left high parasternal view. (c, f) Computed tomographic pulmonary angiography shows a dilated pulmonary artery (PA) but no artifacts. (d) Color Doppler imaging with patent ductus arteriosus flow. (e) Near-suprasternal aortic short-axis view. A linear artifact is clearly seen ((a, b, d, e): white arrowheads). The thymus gland is seen ((a, b, e, f): red arrowheads).

*Case 1*. A 5-year-old boy was diagnosed with PDA and cardiac dilation. A clear diagonal line was seen in the main pulmonary artery. Its shape and length changed in the different echocardiographic views (Figures 1(a), 1(b), 1(d), and 1(e)). A linear line was seen in color Doppler imaging (Figure 1(d)). We also observed the artifact in the patient's video recording (Clip 1). This patient's diagnosis was confirmed by CTPA (Figures 1(c) and 1(f)) and surgery.

**Figure 2:**
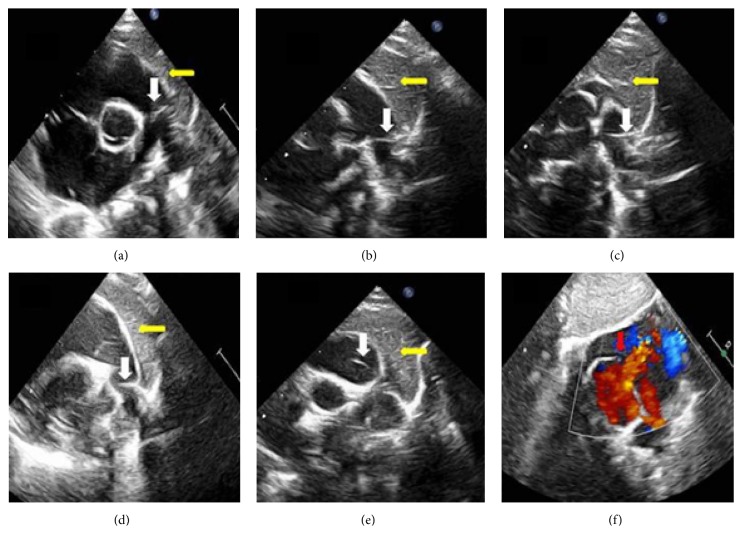
(a) Aortic short-axis view. (b, c) Left high parasternal view. (d, e) Aortic short-axis and pulmonary artery long-axis views. (f) Color Doppler imaging in the subxiphoid two-atrium view. A linear artifact was clearly seen (white arrowhead). The thymus gland was prominently seen (yellow arrowhead).

*Case 2*. A 1-year-old girl was diagnosed with an atrial septal defect (ASD) without pulmonary artery dilation or PAH. A horizontal line was seen in the main and left pulmonary arteries and varied in different echocardiographic views (Figures 2(a)–2(e)). We also visualized the ASD flow from the left to right atrium (Figure 2(f)). The length of the linear artifact was short. The thymus gland was prominently seen. This patient's diagnosis was proven by surgery. The surgeon repaired the ASD and found no fibrous band or dissection in the pulmonary artery.

**Figure 3:**
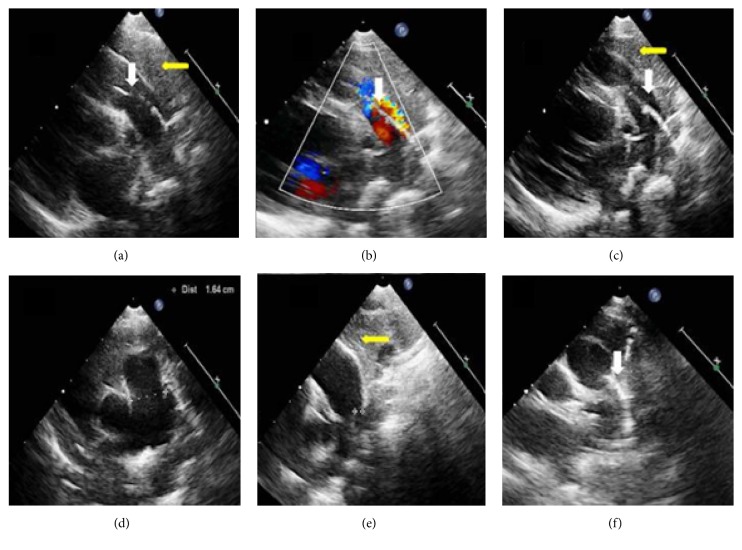
(a-c) Left high parasternal view. (d) Pulmonary artery long-axis view. (e, f) Near-suprasternal aortic short-axis view. (e) Location of the patent ductus arteriosus (white asterisks). A linear artifact is clearly seen (white arrowhead). The thymus gland is seen (yellow arrowhead).

*Case 3*. A 2-year-old girl was diagnosed with PDA. A very long diagonal line was seen in the main pulmonary artery from the left high parasternal view and suprasternal aortic short-axis view (Figures 3(a)–3(c), and 3(f)), but not in the aortic short-axis view or pulmonary artery long-axis view (Figures 3(d) and 3(e)). The long, thick artifact line was clear and could still be seen in color Doppler imaging (Figure 3(b)). The adjacent thymus gland was clearly visualized. This patient underwent PDA ligation without evidence of dissection in the pulmonary artery.

**Figure 4:**
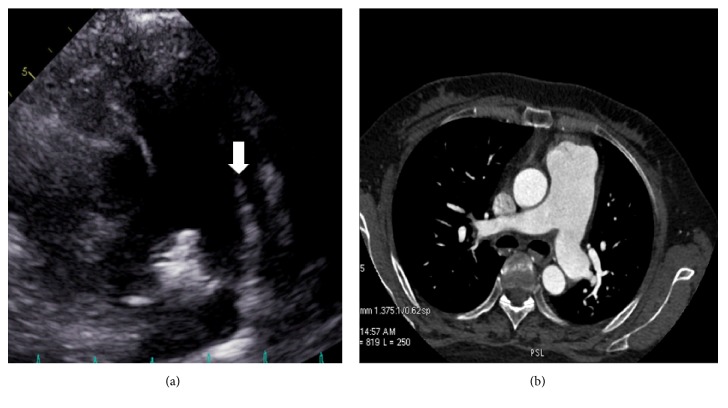
(a) Aortic short-axis and pulmonary artery long-axis views. A linear artifact is clearly seen (white arrowhead). (b) Computed tomographic pulmonary angiography shows that the main pulmonary artery and left and proximal right pulmonary arteries are clear with no artifacts.

*Case 4*. A 38-year-old woman was diagnosed with systemic lupus erythematosus and PAH. Pulmonary artery dilation and a long diagonal line were seen from the left pulmonary artery to the main pulmonary artery (Figure 4(a)). CTPA showed pulmonary artery dilation without a luminal filling defect (Figure 4(b)). The right heart was significantly enlarged and hypertrophied. We also observed the artifact in the patient's video recording (Clip 2).

## 4. Discussion 

Imaging artifacts are frequently encountered in clinical echocardiography. These artifacts may produce different reflections and scatterings when echo is transmitted into different tissue structures. Some common artifacts should be recognized to avoid misdiagnosis. Echocardiographic artifacts are mainly related to wave reflection and/or refraction, such as reverberation, acoustic shadowing, and mirror artifact. Others may be related to ultrasound beam properties and equipment, such as side lobe artifact, beam width artifact, and near-field clutter. Some Doppler artifacts may also be present, such as aliasing, pseudoflow, blooming, and spectral Doppler mirroring.

Based on our review of the literature, echocardiographic artifacts yield a significant number of false-positive results, particularly in the ascending aorta where they appear as aorta intimal flaps [12]. In one study, 55% of patients with clinically suspected aortic dissection had artifacts that needed to be distinguished from a true intimal flap [13]. In another study, most artifacts parallel to the aortic wall appeared as faint linear images in the center of the lumen with slight longitudinal extension to the ascending aorta [14]. Transesophageal echocardiography has high specificity in the diagnosis of aortic dissection, but echocardiographic artifacts can potentially reduce the specificity of transesophageal echocardiography [15]. When applying color Doppler, artifacts do not usually interrupt the blood flow pattern as would be seen with the presence of a true and false lumen. Evangelista et al. [13] described the utility of M-mode to recognize artifacts in 132 patients with suspected aortic dissection by transesophageal echocardiography.

Pulmonary artifacts, similar to those in the ascending aorta, may present as possible dissection flaps [6, 16], which can be a fatal complication of PAH and cardiogenic shock or a cause of sudden death. Pulmonary imaging artifacts may be found in adults with chronic PAH and children with congenital cardiovascular anomalies, particularly PDA and pulmonary artery dilation. We also know that pulmonary artery dissection is very rare, [17] while pulmonary imaging artifact is far more common. Imaging artifacts can change with different ultrasonic views and even extend outside the pulmonary artery or heart. Pulmonary imaging artifacts are seen mainly in the left high parasternal view, which is why many echocardiographers readily miss this type of artifact during a scan; however, sometimes these artifacts can also be found in the regular ultrasonic view. Color Doppler imaging may help to differentiate artifacts from pulmonary artery dissection because artifacts do not usually interrupt blood flow as would be seen with the presence of a true and false lumen (see also Table 2). The origins of echocardiographic imaging artifacts are complex, and their mechanisms are difficult to explain. The most common imaging artifacts encountered in clinical practice are due to the physics of reflection and refraction or to ultrasound beam properties and equipment [5]. Both the beam width and side lobe remain important sources of echocardiographic image artifacts. The side lobe artifact is a common cause of linear artifacts. A small portion of ultrasound energy emitted in “side lobes” is mostly dissipated in the tissue without relevant reflections. However, when this side lobe energy is reflected by a strong reflector (wires, calcifications, pericardium, and other structures) in its path, these reflections are interpreted by the scanner as originating from the central beam. [18] This can produce a linear arc-like artifact at a radial distance from the transducer. This might also be one cause of pulmonary artery imaging artifacts. Another important reason is the effect of reflection between the thymus gland and pulmonary artery (Figures 1–3). This reflection can explain why imaging artifacts are mostly seen in children; we can see clear artifacts from the thymus gland. Another explanation for this type of artifact is the presence of other overlap artifacts from an adjacent structure such as the ascending aorta, especially horizontal short artifacts.

Sometimes CT angiography may be necessary to confirm the diagnosis. However, CT angiography still has some pitfalls and artifacts in patients with suspected pulmonary embolism and dissection, such as improper bolus timing, and streak artifacts, as well as patient-related factors such as motion artifacts, pulmonary arterial catheters, and vascular shunts [6, 19, 20]. It is critically important not to refer patients to cardiac surgery if there is any concern regarding the presence of imaging artifacts.

Recognition of the above-described artifacts in the pulmonary artery has clinical relevance with respect to avoiding misdiagnosis and improving diagnostic accuracy. Rarely, we must differentiate pulmonary artery dissection from artifact to avoid an unnecessary operation.

### 4.1. Limitations

This study is limited by its performance at a national center highly specialized in cardiac imaging and surgery. We hope that our findings will be confirmed by other centers in the future.

## 5. Conclusion

Echocardiography is the diagnostic method of choice to evaluate cardiac anatomy and pathophysiology. Pulmonary artery imaging artifacts simulating dissection flaps can be seen in children with PDA and adults with PAH. An understanding of the types and origins of ultrasound artifacts is important to avoid a false-positive diagnosis of pulmonary artery dissection.

## Figures and Tables

**Table 1:** Demographic data and artifact findings of patients in the present study.

Patient No.	Age (yrs)	Sex	Clinical diagnoses	artifacts					Dilation PA	PH
				view	location	length	direction	distinct		
1	1	F	PDA	1,2	LPA, MPA	long	D	Y	Y	N
2	6	F	PDA	4	MPA	short	H	N	N	N
3	3	F	PDA	1,2	LPA, MPA	long	D	Y	N	N
4	2	F	VSD, PDA	1,2	MPA	long	D	N	Y	N
5	5	M	PDA	1,2,3,4	LPA, MPA	long	D	Y	Y	N
6	0.8	F	PDA	2	MPA	short	D	Y	N	N
7	2	F	PDA	2,3	MPA	long	D	Y	N	N
8	1	F	ASD	1,2	LPA, MPA	short	H	Y	N	N
9	2	F	PDA	1,2,3,4	LPA, MPA	long	D	Y	Y	N
10	37	F	SLE, PAH	1,2	LPA, MPA	Long	D	Y	Y	Y

F: female; M: male; PDA: patent ductus arteriosus; VSD: ventricular septal defect; ASD: atrial septal defect; PS: pulmonary stenosis; PAH: pulmonary artery hypertension; SLE: systemic lupus erythematosus; LPA: left pulmonary artery; MPA: main pulmonary artery; 1: aortic short-axis and pulmonary artery long-axis views; 2: left high parasternal view; 3: suprasternal aortic short-axis view; 4: pulmonary artery short-axis view; D: diagonal; H, horizontal; Y: yes; N: no

**Table 2:** Differences between pulmonary imaging artifact and pulmonary artery dissection.

Baseline data	Pulmonary artifact	pulmonary artery dissection
Incidence rate	high	rare
age	mostly children	mostly adults
Medical history	Mostly seen in PDA	chronic IPAH
Pulmonary pressure	normal or little high	high
Dilation of PA	normal or little wide	Yes
Echo view	mainly left high parasternal	Mainly aortic short-axis and pulmonary artery long-axis view
Location	changing with views	Fixed
CDFI	No interruption	Interruption with flow

PA: pulmonary artery; PDA: patent ductus arteriosus; IPAH: idiopathic pulmonary artery hypertension; CDFI: color Doppler flow imaging

## Data Availability

The data used to support the findings of this study are included within the article.

## Supplementary Materials

Supplementary Materials A clear diagonal line was seen in the main pulmonary artery in a 5-year-old boy diagnosed with PDA. CDFI show a linear artifact but do not interrupt blood flow in the same case. A linear artifact can be seen in a 38-year-old woman diagnosed with systemic lupus erythematosus and PAH.
